# Histological Changes Associated with the Graft Union Development in Tomato

**DOI:** 10.3390/plants9111479

**Published:** 2020-11-03

**Authors:** Carlos Frey, José Luis Acebes, Antonio Encina, Rafael Álvarez

**Affiliations:** 1 Departamento de Ingeniería y Ciencias Agrarias, Área de Fisiología Vegetal, Facultad de Ciencias Biológicas y Ambientales, Universidad de León, 24071 León, Spain; jl.acebes@unileon.es (J.L.A.); a.encina@unileon.es (A.E.); 2 Departamento de Biología Molecular, Área de Biología Celular, Facultad de Ciencias Biológicas y Ambientales, Universidad de León, 24071 León, Spain; ralvn@unileon.es

**Keywords:** callus, grafting, histology, rootstock, scion, *Solanum lycopersicum*

## Abstract

Despite the importance of grafting in horticultural crops such as tomato (*Solanum lycopersicum* L.), the structural changes that occur during the graft establishment are little understood. Using histological techniques, the present work examines the time course of changes on the anatomical structure of the graft junction in functional tomato homografts and compares it to that of heterografts and non-functional grafts. No apparent differences were detected between homo- and heterografts, showing similar tissue development. At 10 days after grafting, the cell walls of the scion and rootstock in the area of the graft junction were thicker than usual. Undifferentiated cells and new vascular tissue emerged from the pre-existing vasculature. Adventitious roots appeared mainly on the scion, arising from the pre-existing vasculature. At 20 days, more pronounced vascular tissue was visible, along with large areas showing vascular connection. At 210 days, vestiges of the changes undergone in graft development were still visible. Generally, non-functional grafts presented layers of necrotic remains and deposition of cell wall material in the cut edges, impeding the suitable scion-rootstock connection. Our results show that accurate changes in pre-existing vasculature and the cell walls of the adhesion line are crucial to the development of functional grafts.

## 1. Introduction 

The regenerative properties of plants have been harnessed by growers since ancient times to graft together specimens of different varieties or even species [1]. A graft is composed of a root-bearing portion (the rootstock) and an aerial portion (the scion) provided by two plants [2]. Grafting is now performed worldwide and is of great economic importance [3,4,5,6]. Grafting has been revealed as a very useful technique in order to improve resistance to disease, insects and abiotic stress, to improve yields, to control plant size, to shorten the juvenile period, as a method of propagation, to induce unusual forms of growth, and to provide models for studies of plant physiology [1,2,7,8]. 

Many complex biochemical and structural processes take place during the establishment of a graft, from the response to the initial wound, changes at the edges of the cut, the formation of callus tissue, the differentiation of new vascular tissue, and the connection of the vasculature [2,9,10]. All these processes determine the success of a graft; understanding them, their timing, and where they occur, is key in understanding and promoting graft establishment.

Grafting is widely used with tomato (*Solanum lycopersicum* L.) [5], one of the most economically important crops in the world [3,5]. The advantages that grafted plants offer in terms of productivity render the process indispensable to many growers. The literature contains little information on the precise structural development of graft junctions in this or any other crop. Moreover, a better understanding of structural and molecular mechanisms underlying graft formation might help improve the success and quality of grafting to increase the economic outcome of this technique. Within this frame, the aim of the present work was to record the sequence of histological modifications that occur over time in the graft junction of tomato functional homografts, as a model of good scion-rootstock compatibility, to compare it with that of intraspecific heterografts, as a model of moderate compatibility and with that of non-functional grafts.

## 2. Results

**Figure 1:**
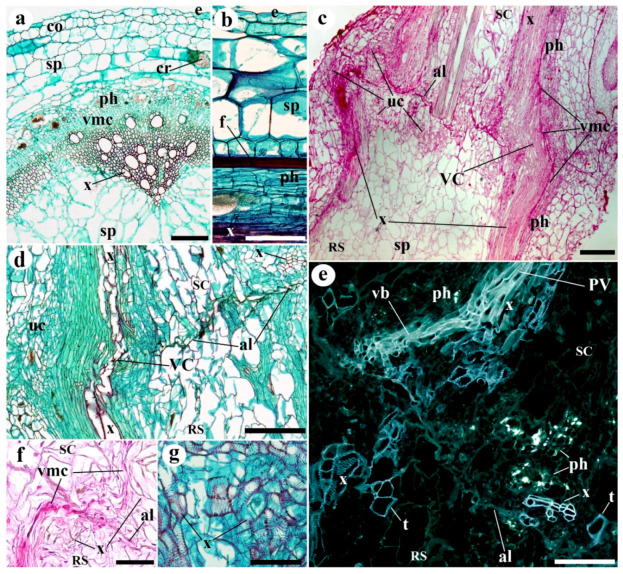
Representative images of stem sections of (**a**,**b**) 0 (non-grafted) and (**c**–**g**) 10 days after grafting (10 DAG) plants. (**a**)Transverse section of a stem of *Solanum lycopersicum* (Minibel) showing the distribution of the tissues during primary growth. (**b**) Longitudinal section of a stem; note the distribution of tissues from the exterior to interior (up-down). (**c**) Longitudinal section of the graft junction area; note the adhesion line (al), the new vascular connections (VC), and the areas with undifferentiated cells (uc). (**d**) Longitudinal section of the graft junction area; note the vascular connections (VC) and adhesion line (al). Close to the adhesion line, in the scion, note the groups of xylem cells (x). (**e**) Longitudinal section of the graft junction area; note the transdifferentiated parenchyma cells (t) in the scion and rootstock, the phloem (ph, yellow points), the vascular branch (vb) arising from the pre-existing vasculature (PV), and the large number of neo-differentiated cells in both scion and rootstock. (**f**) Longitudinal section of the graft junction area; note the vascular connection between the scion and rootstock, and how the vascular meristematic cells make contact across the adhesion line (al). (**g**) Differentiation of conducting xylem cells within callus tissue. (**a**,**b**,**d**,**g**) Safranin-fast green; (**c**,**f**) Haematoxylin-Eosin; (**e**) Sirofluor. (**a**–**d**,**f**,**g**) Bright field view. (**e**) Epifluorescence microscopy. *al* adhesion line, *cr* microcrystals, *co* collenchyma, *e* epidermis, *f* fiber, *PV* pre-existing vascular tissue, *ph* phloem, *RS* rootstock, *SC* scion, *sp* storage parenchyma, *t* transdifferentiating cells, *uc* undifferentiated cells, *vb* vascular branch, *VC* vascular connection, *vmc* vascular meristematic cells, *x* xylem. Scale bars: a, d, e = 150 µm; b = 100 µm; c = 300 µm; f = 200 µm; g = 75 µm.

At 5 weeks post-emergence, the non-grafted control plants normally had a green shoot with a very large number of trichomes (data not shown). The tissues identified in the stem included a monolayer of epidermal cells, laminar collenchyma, photosynthetic parenchyma, storage parenchyma (cortical), primary phloem, vascular meristematic tissue, primary xylem and storage parenchyma (medullary) (Figure 1a,b). The vascular system appeared in a ring with clear differentiation between fascicular and interfascicular areas. Phloem fibers as well as microcrystals were also present. 

**Figure 2:**
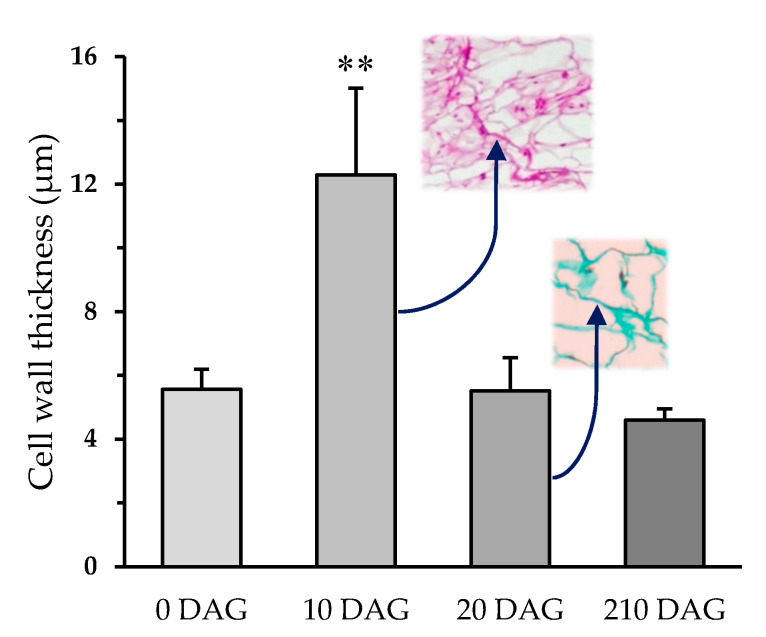
Cell wall thickness (µm) of pith cells in the adhesion line of homografts at different days after grafting (DAG). Average and standard deviation are represented (N = 80). Student *t* test was performed between 0 DAG vs. 10, 20, and 210 DAG; ** *p* < 0.01.

The success of grafting was high in homografts, approximately 85%, and moderate in the two types of heterografts, approximately 60%. The functional homografts and heterografts showed similar tissular changes and no apparent differences between them were detected. In addition, no differences were detected between the development of the two types of heterografts. At 10 days after grafting (DAG), a necrotic line appeared where the stem was cut (Figure S1i,j). Later, this disappeared as the callus grew (it can attain a large size). The histological organization of the graft junction was very different to that of the control stems. At 10 DAG (Figure 1c–f), the tissues of the scion and rootstock are joined. An adhesion line (see *al* in Figure 1c–f) formed by thickened walls of cells belonging to the rootstock and scion was distinguishable to a varying extent at this stage of grafting (Figure 2). The remains of necrotic tissue and areas of non-adhesion were also visible. The rootstock and scion tissues were often interdigitated. Masses of undifferentiated cells, developing vascular elements, and sometimes vascular connections, were also appreciable. These masses of undifferentiated cells make up most of the callus, and were seen in their largest numbers near pre-existing vascular tissue and in the medullary and cortical parenchyma close to the wound (Figure 1c,d). Vascular cells (phloem and xylem cells) differentiated during the establishment of the graft (Figure 1c–g): (1) appearing as a branch of the pre-existing vasculature; (2) as forming vascular pockets or groupings dispersed throughout the callus; (3) and as cells transdifferentiating from parenchyma into xylem vessels. Particularly, callose labeling was used for accurate following of phloem differentiation (see *ph* in Figure 1e). Generally, the most distal extreme of the pre-existing vasculature remained inert (Figure S2a), playing no active part in the vascular re-connection occurring during grafting. The response of the scion cells seemed to be greater than that of the rootstock in terms of the differentiation of vascular connections. Ruthenium red staining showed an intense deposition of pectic polysaccharides in the cut edges pre-adhesion, then, these polysaccharides remained in the adhesion area of functional grafts (Figure S3b,d). Calcofluor white fluorescence revealed crushed cells and cellulose rich cell walls in the cut edges pre-adhesion; at 8 and 20 DAG, cell wall thickenings were retained in the adhesion line (Figure S3a,c,e).

**Figure 3:**
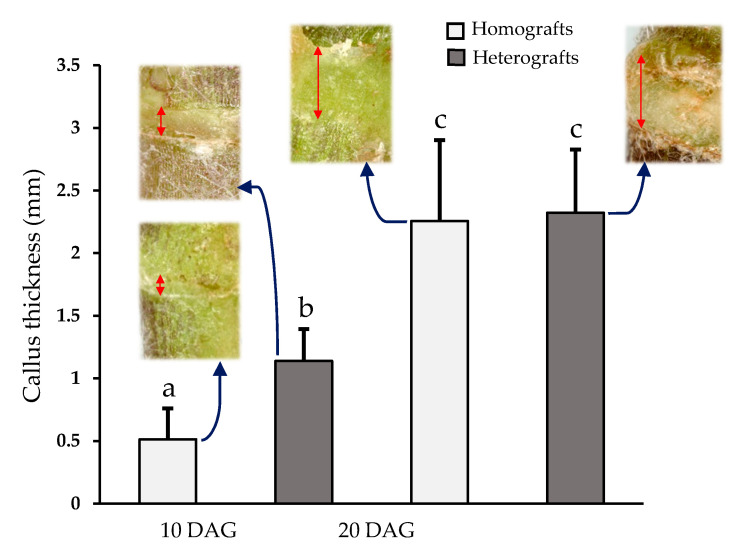
Callus thickness (mm) in homografts ☐ and heterografts ■ at 10 and 20 days after grafting (DAG). Average and standard deviation are represented (N = 12). ANOVA variance analysis with Tukey pos-hoc test was performed; equal letters indicates not significant differences (*p* < 0.05).

**Figure 4:**
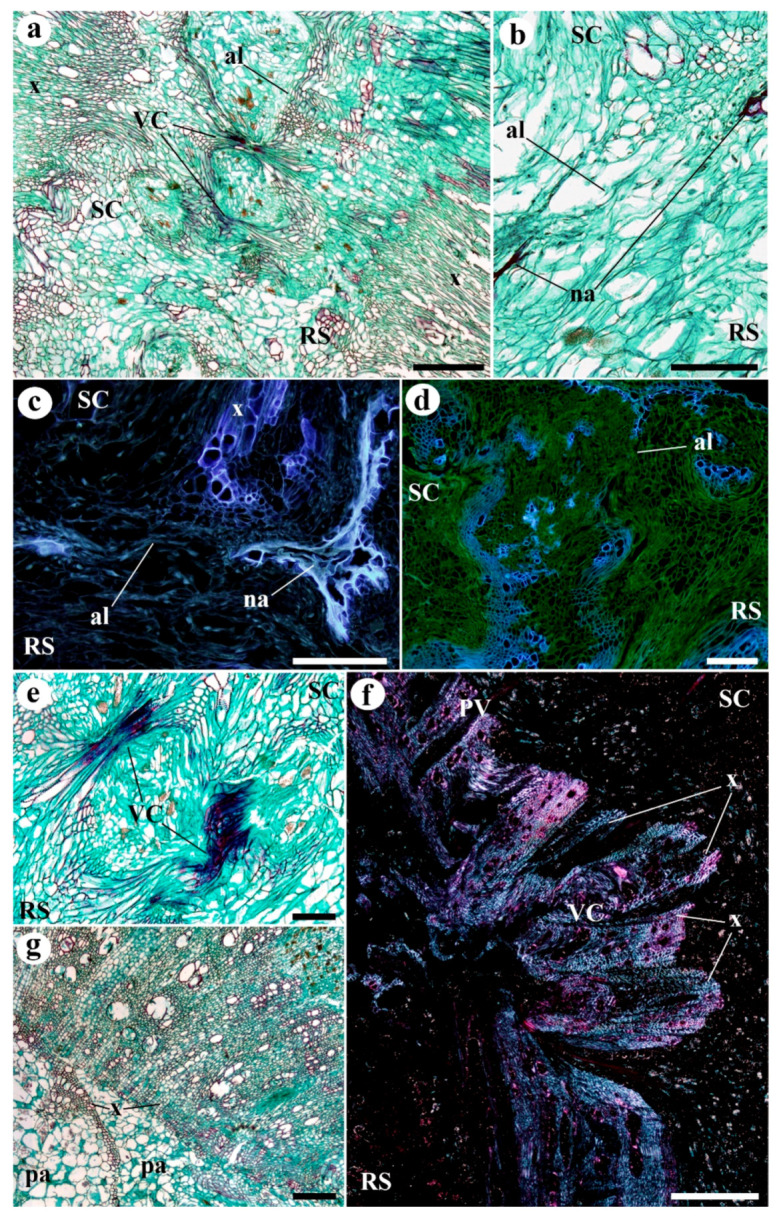
Representative images of (**a**–**c**,**f**,**g**) homografts and (**d**,**e**) heterografts at (**a**–**e**) 20 days and (**f**,**g**) 210 days after grafting (DAG). (**a**) Longitudinal section of the graft junction area. Note the large amount of vascular tissue, mostly xylem (x), and the vascular connections (VC). The adhesion line (al) is no longer so clearly visible. (**b**,**c**) Longitudinal section of the adhesion zone. The adhesion line (al) is hard to distinguish, but areas of non-adhesion (na) are clearly visible. (**d**) Minibel (scion)–Marmande VR (rootstock) heterograft. Longitudinal section of the graft junction area. Note the large amount of vascular tissue, mostly xylem (x). (**e**) Marmande VR (scion)–Minibel (rootstock) heterograft. Longitudinal section of the graft junction. Note the vascular connections detail (VC). (**f**) Longitudinal section of the graft junction. Note the vascular connection (VC) now clearly formed. Note too that the xylem (x) in the junction is different to that seen in the pre-existing vasculature (*PV*). (**g**) Transverse section of the graft junction area, showing the discontinuity of the vascular ring, note the left-over disconnected xylem (x) by parenchyma (pa) tissue**.** (**a**,**b**,**g**) Safranin-fast green. (**a**,**b**,**e**,**g**) Bright field view. (**c**,**d**) Epifluorescence microscopy. (**f**) Polarization microscopy. *al* adhesion line, *na* non-adhesion, *pa* parenchyma, *ph* phloem, *PV* pre-existing vascular tissue, *RS* rootstock, *SC* scion, *VC* vascular connection, *x* xylem. Scale bars a, c, g = 200 µm; b, d, e = 100 µm; f = 300 µm.

The thickness of the callus at the junction zone steadily increased from 10 to 20 DAG (Figure S1). Although at 10 DAG, calluses from heterografts were significantly thicker than those from homografts, this difference was no longer appreciated at 20 DAG (Figure 3). At 20 DAG, the adhesion line was almost imperceptible (Figure 4a–d). By this stage in tomato grafting, the wall thickness of pith cells at the junction zone significantly reduced when compared with 10 DAG. This later parameter remained unchanged for longer times (see *210 DAG* in Figure 2). Some areas of non-adhesion persisted. Hyperplasia and hypertrophy were observed at this stage in the graft junction (Figure 4a,d). The callus in the junction area contained many differentiated vascular cells—more than any other type (Figure 4a–e). Vascular connections were complete (Figure 4a,e). The outer callus tissue of the scion formed a ‘skirt’ containing vascular tissue surrounded by parenchyma (Figure S2). 

At 210 DAG (Figure 4f,g), the graft junction looked similar to that seen at 20 days. However, the vascular connections were better defined, and the tissues were arranged in a more orderly fashion. Vestiges of the process of union between the scion and rootstock were visible in transverse sections (Figure 4g and Figure S2c,d). 

**Figure 5:**
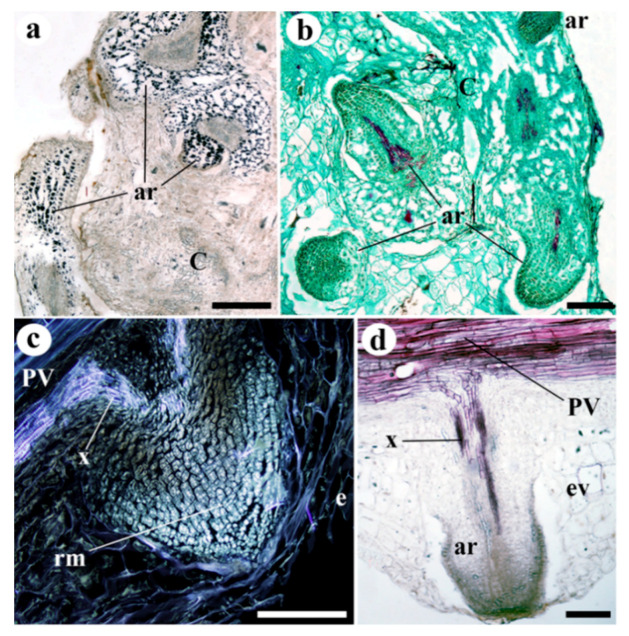
Adventitious roots arising from the scion at 20 days after grafting. (**a**) Longitudinal section of an adventitious root (ar); note the many amyloplasts (black dots). (**b**) Adventitious roots (ar) arising from the callus (C) of the scion near the graft junction; note how the top adventitious root breaks through the epidermis. (**c**) Longitudinal section of an adventitious root primordium, showing how it arises from the pre-existing vasculature (PV); the root meristem (rm) is also distinguishable. Note how the epidermis is perforated (**e**). (**d**) Longitudinal section of an adventitious root (ar) primordium, showing how the root vascular tissue arises from the pre-existing vasculature (PV). Note the enveloping parenchyma around the structure of the root (ev). (**a**) Lugol; (**b**) Safranin-fast green; (**d**) Phloroglucinol. (**a**,**b**,**d**) Bright field view. (**c**) Epifluorescence microscopy. *ar* adventitious root, *C* callus, *e*, epidermis, *ev* enveloping parenchyma, *PV* pre-existing vascular tissue, *rm* root meristem, *x* xylem. Scale bars: a = 300 µm; b = 200 µm; c, d = 100 µm.

At 10 and 20 DAG, adventitious roots appeared sometimes on the scion (Figure 5a–d and Figure S1), but these were gone by 210 days. They were only seen in that part of the scion close to the graft junction, arising from the meristematic tissue of the pre-existing vasculature and growing between callogenic tissue. In addition, at 20 DAG, a parenchyma envelope was detected in the adventitious roots. Generally, the cells of these adventitious roots contained many amyloplasts.

**Figure 6:**
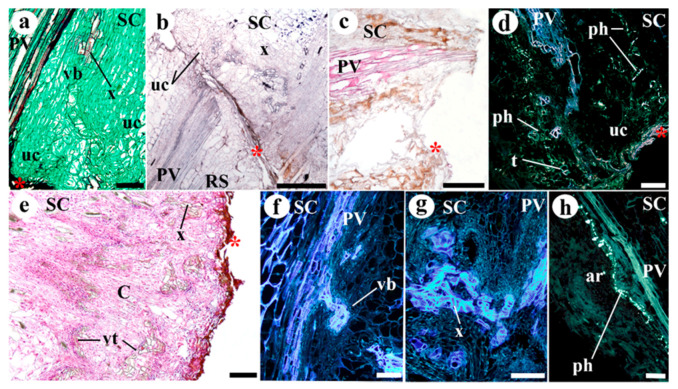
Representative images of non-functional (**e**) homografts and (**a**–**d**,**f**–**h**) heterografts at (**a**–**d**) 10 and (**e**–**h**) 20 days after grafting (DAG). (**a**) Marmande VR (scion)–Minibel (rootstock) heterograft. Longitudinal section of cut edge close section; note the undifferentiated cells (uc) and vascular branch (vb) arising from the pre-existing vasculature (PV). (**b**) Minibel (scion)–Marmande VR (rootstock) heterograft. Longitudinal section of non-functional graft union; note the layers of necrotic remains and deposition material in the cut edges of the scion and rootstock. In addition, see the undifferentiated cells (uc) and new xylem (x). (**c**) Minibel (scion)–Marmande VR (rootstock) heterograft. High accumulation and deposition of necrotic remains and deposition material in the scion of non-functional graft; note the pith destruction. (**d**) Marmande VR (scion)–Minibel (rootstock) heterograft. Longitudinal section of cut edge area; note the transdifferentiated parenchyma cells (t), the undifferentiated cells (uc), and the phloem (ph, yellow points). (**e**) Longitudinal section of scion cut edge; note the presence of large number of new-differentiated cells that form the callus tissue. Additionally, see the layers of necrotic remains and deposition material in the cut edge. (**f**,**g**) Minibel (scion)–Marmande VR (rootstock) heterografts. Vascular differentiation close to the cut edge of scion; note the new-vasculature differentiation, especially, note the vascular branch (vb) arising from the pre-existing vasculature (PV) in g. (**h**) Minibel (scion)–Marmande VR (rootstock) heterograft. Adventitious root (ar) of non-functional graft, note that it arises from the pre-existing vasculature (PV). (**a**) Safranin-fast green; (**b**) Lugol; (**c**) Phloroglucinol. (**d**,**h**) Sirofluor; (**e**) Haematoxylin-Eosin. (**a**, **b**,**c**,**e**) Bright field view. (**d**,**f**,**g**,**h**) Epifluorescence microscopy. Asterisk design necrotic remains and material depositions layers in the cut edges. *ar* adventitious root, *C* callus, *ph* phloem, *PV* pre-existing vascular tissue, *RS* rootstock, *SC* scion, *t* transdifferentiating cells, *uc* undifferentiated cells, *vb* vascular branch, *vt* vascular tissue, *x* xylem. Scale bars: a, g, h, i = 100; b, c = 500 µm; d, e = 200 µm.

Non-functional grafts presented a similar visual and histological response, independently of the type of combination (homografts or heterografts). Non-functional grafts did not present graft union or scion-rootstock adherence, but often adventitious roots on the scion appeared (Figure S1k). The cut edges were sealed by layers of necrotic remains and deposition materials with variable thickness (Figure 6a–e). Generally, undifferentiated callus cells and new vasculature was detected at 10 and 20 DAG. As in functional graft development, undifferentiated cells (at 10 DAG) made up most of the callus and were particularly abundant near pre-existing vascular tissue and in the medullary and cortical parenchyma close to the wound (Figure 6a,b,d–h). In addition, the three different patterns of vascular cell differentiation were detected: as a branch of the pre-existing vasculature, as vascular pockets within the callus and as transdifferentiated cells (Figure 6a,b,d–h). Adventitious roots often appeared in non-functional grafts, they were only seen in the part of the scion close to the graft junction and arising from the meristematic tissue of the pre-existing vasculature as in functional grafts (Figure 6i).

Table 1 summarizes most of the above observations.

## 3. Discussion

The distribution of the tissues in the scion and rootstock is essential in the correct establishment of the graft, in particular, the vascular meristematic tissue [2,11]. The stems of the tomato have the typical structure of dicotyledonous, mesophytic plants, with a vascular cylinder that appears discontinuous in the early stages of life, but which later becomes continuous with the interfascicular regions [12,13]. This distribution allows for grafting, unlike the dispersed vascular pattern of monocotyledonous plants [2,10,14].

Although a difference in the success rate comparing Minibel homografts and Minibel-Marmande VR heterografts was found in our study, homografts and heterografts did not show ostensible histological differences, regardless of whether functional or non-functional grafts were considered. This lack of differences would be related to the fact that both varieties share a high genetic similarity [15]. Therefore, it would be interesting in the future to study histological differences among heterografts formed by combination of plants with a progressive lower genetic similarity, beginning with those belonging to the same Lycopersicon group (which comprise the domesticated tomato *Solanum lycopersicum* and the most close species such as *S. cheesmaniae*), then to species from other groups of the *Solanum* section *Lycopersicon*, as *Arcanum*, *Neolycopersicon*, or *Eriopersicon* groups, after that to other sections of the genus *Solanum* and finally to other Solanaceae species [16,17]. Then, probably can be shown that the larger the genetic distance between the partners of the grafts, the higher dissimilarity regarding the histology of the graft junction. 

The cell walls at the line of contact between the scion and rootstock commonly appeared thickened. These structures are thought to play a key role in graft establishment, taking part in the recognition and adhesion of the two sides of the graft [18,19,20,21,22,23,24]. The increase in thickness is related to the deposition of wall polysaccharides by the protoplasts of nearby cells and the compacting of necrotic remains from the cut [25]. In our work, cellulose and pectin deposition was detected in these thickenings, and particularly, this latter type of polysaccharides would take part in the recognition and make the first adhesion. The later thinning of these walls is accompanied by the restitution of the symplastic pathway, owing to the formation of plasmodesmata [18,21,25] and the clearing of cell wastes [7,26].

The tissues close to the cut also undergo profound changes [2,10]. In tomato, the callus is large, but this would not appear to have a bearing on the success of the graft; in other dicotyledonous species, the callus can be very small (in some cases almost imperceptible), yet grafting is successful [26,27].

The callus is generated by the de-differentiation of cells close to the wound [10,28,29], but it is hard to say which types are most involved. In the present work, the location and appearance of the cells and tissues pointing to the callus is formed largely from cells associated with the vascular tissue. This might be related to the preferential expression of the transcription factor *WIND1* by the vascular meristematic tissue [29]. *WIND1* is involved in de-differentiation and callus formation via the activation of a signaling pathway in which cytokinins participate [29,30,31]. Auxins also have the capacity to induce callus formation [28], although in graft establishment their induction of vascular differentiation is likely more important [32,33,34,35,36,37]. These hormones, which travel in a basipetal direction (i.e., downward from the tip of the scion), move through the stem, transported by PIN-FORMED (PIN) transport proteins [38]. These transporters can alter their position in the cell surface towards wounds [39,40]. Their accumulation in the scion vasculature, provoked by the cut, would seem to induce nearby cells to become meristematic. The differentiation of new vasculature occurs in different directions, away from the pre-existing vascular tissues, and these new branches may connect with pockets of vasculature forming in the callus. As reported in other studies [18,25,41], vascular connections between the rootstock and scion are clearly visible at around 10 DAG. The connection of the scion and rootstock tissues leaves a permanent mark at the graft junction, especially in the vascular tissue. 

The formation of adventitious roots by the scion is a response to the stress caused by the wound [42,43]. The process is directly related to the blockage of the basipetal movement of auxins at the cut and their accumulation in that area too [44,45]. The production of these roots is not helpful in graft establishment, indeed, rooting could prevent the graft being successful [45]. However, it could be useful in nurse-rooting [2,44]. Sala et al. [43] identified two types of adventitious root, produced at different times after grafting: one with and one with no parenchyma envelope. In this work, we detected adventitious roots with parenchyma envelope at 20 DAG. 

Non-functional grafts (regardless of whether homografts or heterografts) lacked the ability to develop a complete graft union, mainly due to the presence of necrotic remnant which prevent the progress of the correct sequence of events that characterize the functional graft. In some cases, adherence between rootstock and scion appeared but it is likely that the deposition of a large amount of cell wall materials sealed the cut edges and prevent the exchange of substances. Lignin and/or suberin have been frequently suggested to be associated to wound responses [46,47] that could impede the development of functional grafts [47]. In addition to this, other processes associated to the sequence of facts, such as the formation of callus constituted by undifferentiated cells, the differentiation of vascular cells, as well as the formation of adventitious roots were observed, pointing to that the unfunctionality of the grafts would be associated to an exacerbated defense response associated to cell walls in the cut edge. These results point to the role of cell wall in the cut edge of unfunctional grafts deserve a more in-depth study in order to a better understanding of the graft unfunctionality and incompatibility.

In summary, our study shows that the graft junction (whether in homografts or heterografts) undergoes modifications essential for adequate functioning of the grafted plant. The early stage of graft establishment in tomato is marked by thickened cell walls at the graft junction. The pre-existing vasculature plays an important role producing callus tissue and new vascular cells. By 10 days post grafting, some areas of xylem and phloem of the scion and rootstock are connected and functional. The scion vasculature is also the source of adventitious roots. Long after the graft is established, vestiges of the process of joining remain apparent. However, in non-functional grafts, although most of the events associated to the graft unio /n, such as the formation of callus constituted by undifferentiated cells and the differentiation of vascular cells, are present, the sealing of cut edges with the deposition of cell wall materials prevents the correct progression of the graft formation. 

## 4. Material and Methods 

### 4.1. Plants and Growth Conditions

Cherry tomato (Minibel) seeds (Mascarell Semillas SL) and RAF tomato (Marmande VR) (Semillas Batlle SA) were sown individually in 200 mL plastic pots containing 170 ± 10 mL of peat, placed on plastic trays. Watering with complete Hoagland nutritive solution, directly into the trays, was performed twice per week. The plants were grown in a growth chamber maintained at 23 ± 1 °C and with a 16 h light period.

### 4.2. Grafting Method and Healing Conditions

Grafting was undertaken when the plants stems were 4–5 mm thick (after 5 weeks of growth). Minibel homografts (scions and rootstocks from the same variety), Minibel (scion)–Marmande VR (rootstock) heterografts, and Marmande VR (scion)–Minibel (rootstock) heterografts were performed. Non-grafted (0 DAG) plants were used as controls. Thirty plants were prepared for every experimental condition. Scions and rootstocks were obtained by making a transverse cut below the cotyledon leaves. Cut faces were then joined, and the junctions wrapped in Parafilm^®^ and protected using Toogoo^®^ silicon clips. The grafted plants were kept for 7 days in a growth chamber at 23 ± 1 °C, with a 16 h light period (≈35 µmol m^−2^ s^−1^) and with humid atmosphere (≈ 90% RH), which was afterwards gradually ventilated. Success of grafting (*n* = 30 for each experimental condition) was calculated by morphological observation of union between rootstock and scion (see Figure S1).

### 4.3. Post-Healing Cultivation

After graft healing, the long-time grafts (210 DAG) were grown in a growth chamber maintained at 23 ± 1 °C and with a 16 h light period and watering with Hoagland nutritive solution, directly into the trays, was performed twice per week.

### 4.4. Cell Wall Thickness

Pith cells wall thickness in the adhesion line was measured at different DAG. One measure was reported per cell. Measurements of cell wall thickness were taken with ImageJ 1.52a software.

### 4.5. Callus Thickness

At 10 and 20 DAG, grafts were observed with a stereoscopic microscope (Nikon 105 SMZ 1500) and photographed using a Nikon D4 full-frame camera and NIS-Elements F.3.2 106 software. The measures of callus thickness were taken with ImageJ 1.52a software.

### 4.6. Histological Techniques

Segments containing the graft junction (or the equivalent stem in 0 DAG–non-grafted plants) were taken from successfully grafted plants at 10, 20, and 210 DAG, and from unsuccessfully grafted plants at 10 and 20 DAG (*n* = 5 per graft type and time point). Samples were fixed in formalin–acetic acid–alcohol (FAA) (24–48 h), before placing in increased ethanol series, isoamyl acetate, and finally embedded in paraffin wax. A rotary microtome was then used to make 12 µm sections which were stained with either safranin and fast green, hematoxylin and eosin, lugol, phloroglucinol, sirofluor (a fluorescent marker) or ruthenium red. After mounting (see Table 2 for these and other details), slides were observed under bright field, polarized and epifluorescence (UV-2 filter) conditions using a Nikon E600 microscope. Twenty-five slides with 2–4 sections for every experimental condition and time were prepared, and 5–10 fields were observed for each section in every slide. In order to obtain sections for cellulose detection, the samples were fixed in 2.5% (*w/v*) *p*-formaldehyde in 0.1 M phosphate buffer pH 7.5 at 4 °C overnight. The fixed tissues were dehydrated in decreasing ethanol series and embedded in resin (LR-White, London Resin, Reading, UK). The embedded samples were placed in gelatin capsules with resin and then incubated at 37 °C for 5 days to polymerize. Ultracut Microtome LKB 2088 (Reichart Jung^®^, Wien, Austria) was used to obtain semithin (1 µm) longitudinal sections. The sections were placed in slides coated with Vectabond^®^ reagent (Vector Laboratories, Burlingame, CA, USA) and cellulose staining was performed using 0.005% (*w/v*) calcofluor white (fluorescent-brightener 28, Sigma^®^, St. Louis, MO, USA) (see Table 2). Nikon E600 epifluorescence microscope with the UV-2 filter was used for the observation.

### 4.7. Statistical Analyses

Cell wall and callus thickness differences were analyzed by Student *t* test and ANOVA variance analyses. They were performed by statistical software SPSS *v*.25 (SPSS Federal Systems, Chicago, IL, USA); *p*-values < 0.05 were considered to be statistically significant.

## Figures and Tables

**Table 1:** Summary of histological characteristics corresponding to the graft junction of functional and non-functional homografts and heterografts in *Solanum lycopersicum* at 10, 20, and 210 days after grafting. No apparent differences among homografts and heterografts were observed.

Graft Type	Functional Grafts			Non-Functional Grafts	
Days after grafting	10	20	210	10	20
Necrotic remnant	+	−	−	+ +	+ + +
Areas of non-adhesion	+	+	+	+ + +	+ + +
Undifferentiated cells	+ +	+	−	+ +	+
Xylem cells	+	+ + +	+ + +	+	+ + +
Vascular connections	±	+	+	−	−

[+] presence; [−] absence; relative amounts indicated by number of symbol repetitions (subjective description).

**Table 2:** Preparation of slides and microscope observations.

Staining	Mounting	Microscopy Technique	Target
Safranin-Fast Green	Entellan	Bright field	General staining
Hematoxylin-Eosin	Entellan	Bright field	General staining
Lugol	No	Bright field	Starch
Ruthenium red	No	Bright field	Pectic polysaccharides
Sirofluor	No	Epifluorescence	Callose
Phloroglucinol	No	Bright field	Lignin
No	Entellan	Epifluorescence	Autofluorescence
No	Entellan	Polarization	Birefringent structures
Calcofluor White	No	Epifluorescence	Cellulose

## Supplementary Materials

The following are available online at https://www.mdpi.com/2223-7747/9/11/1479/s1, Figure S1: Graft junctions at (a–d) 10 and (e–k) 20 DAG. a, b, e, f, i, k Homograft; c, g, j Minibel (scion)–Marmande VR (rootstock) heterograft; d, h Marmande VR (scion)–Minibel (rootstock) heterograft. Scale bars: a–h, k = 1 mm; i, j = 0,5 mm. *ar* adventitious roots, *c* callus, *RS* rootstock, *SC* scion. In a–h the scion is the top part and rootstock the bottom part. Note the adventitious roots arising on the scion and the changes in the callus development. i, j Callus detail. k Unsuccessfully grafted plant, note the absence of scion–rootstock adhesion; Figure S2: Details of graft histological development. a Pre-existing vascular tissue (PV) at 10 DAG; note how the most distal end is composed of dead cells only. b Longitudinal section of the expansion of the callus (CE) at 20 DAG; note the xylem (x) and the parenchyma (pa) towards the exterior. c and d Vestiges of the graft process at 210 DAG: c Transverse section of the graft junction area, showing the discontinuity of the vascular ring. d Transverse section of the graft junction area; note the anomalies of the vascular ring. a Hematoxylin-Eosin; b–d Safranin-fast green. a–d Bright field view. CE, callus expansion, na non-adhesion, pa parenchyma, ph phloem, PV pre-existing vascular tissue, RS rootstock, SC scion, x xylem. Scale bars: a, d = 100 µm; b, c = 200 µm; Figure S3: Longitudinal sections staining for polysaccharides (calcofluor White -cellulose- and ruthenium red -pectins-) from homografts. a Rootstock section at 2 DAG; note the cut edge, especially the crushed cells and remnants (ccr), as well as the intense fluorescence from cellulose of these cell walls. b Rootstock section at 4 DAG; note the deposition of high amount of pectins at the cut edge. c Early graft union section at 8 DAG; note the line of adhesion with cellulosic cell wall thickenings. d Graft union section at 20 DAG; note the permanence of the pectin depositions at the adhesion line as well as vascular (re)connections (vc). e Graft union section at 20 DAG; note the cellulosic cell walls at line of adhesion and transversal section of vasculature (xylem and phloem). a, c, d Calcofluor White; b, e Ruthenium red. a, c, d Epifluorescence microscopy. b, e Bright field view. ccr crushed cells and remains, pd pectin deposition, ph phloem, RS rootstock, SC scion, VC vascular connections, x xylem. Green asterisk indicates cut edges. Triangle arrows indicate adhesion line. Scale bars: a, c = 50 µm; b, d, e = 100 µm.

## Abbreviations

*al*: adhesion line; *ar*: adventitious root; *C*: callus; *CE*: callus expansion; *co*: collenchyma; *cr*: microcrystals; *e*: epidermis; *ev*: enveloping parenchyma; *f*: fiber; *FAA*: formalin–acetic acid–alcohol; *na*: non-adhesion; *pa*: parenchyma; *ph*: phloem; *PV*: pre-existing vascular tissue; *rm*: root meristem; *RS*: rootstock; *SC*: scion; *sp*: storage parenchyma; *t*: transdifferentiating cells; *uc*: undifferentiated cells; *vb*: vascular branch; *VC*: vascular connection; *vmc*: vascular meristematic cells; *x*: xylem.
