# Supplementary material for: Histological Changes Associated with the Graft Union Development in Tomato
Source: Plants (Basel). 2020 Nov 3;9(11):1479. doi: 10.3390/plants9111479 (PMC7692471; doi:10.3390/plants9111479)
Supplement: Supplementary file 1 [file plants-09-01479-s001.pdf]

plants

MDPI

Article

Histological Changes Associated with the Graft Union Development in Tomato

Carlos Frey [1,*], José Luis Acebes [1], Antonio Encina [1] and Rafael Álvarez [2]

[1] Departamento de Ingeniería y Ciencias Agrarias, Área de Fisiología Vegetal, Facultad de Ciencias Biológicas y Ambientales, Universidad de León, 24071 León, Spain; jl.acebes@unileon.es (J.L.A.); a.encina@unileon.es (A.E.)

[2] Departamento de Biología Molecular, Área de Biología Celular, Facultad de Ciencias Biológicas y Ambientales, Universidad de León, 24071 León, Spain; ralvn@unileon.es (R.A.)

* Correspondence: cfred@unileon.es

Supplementary material

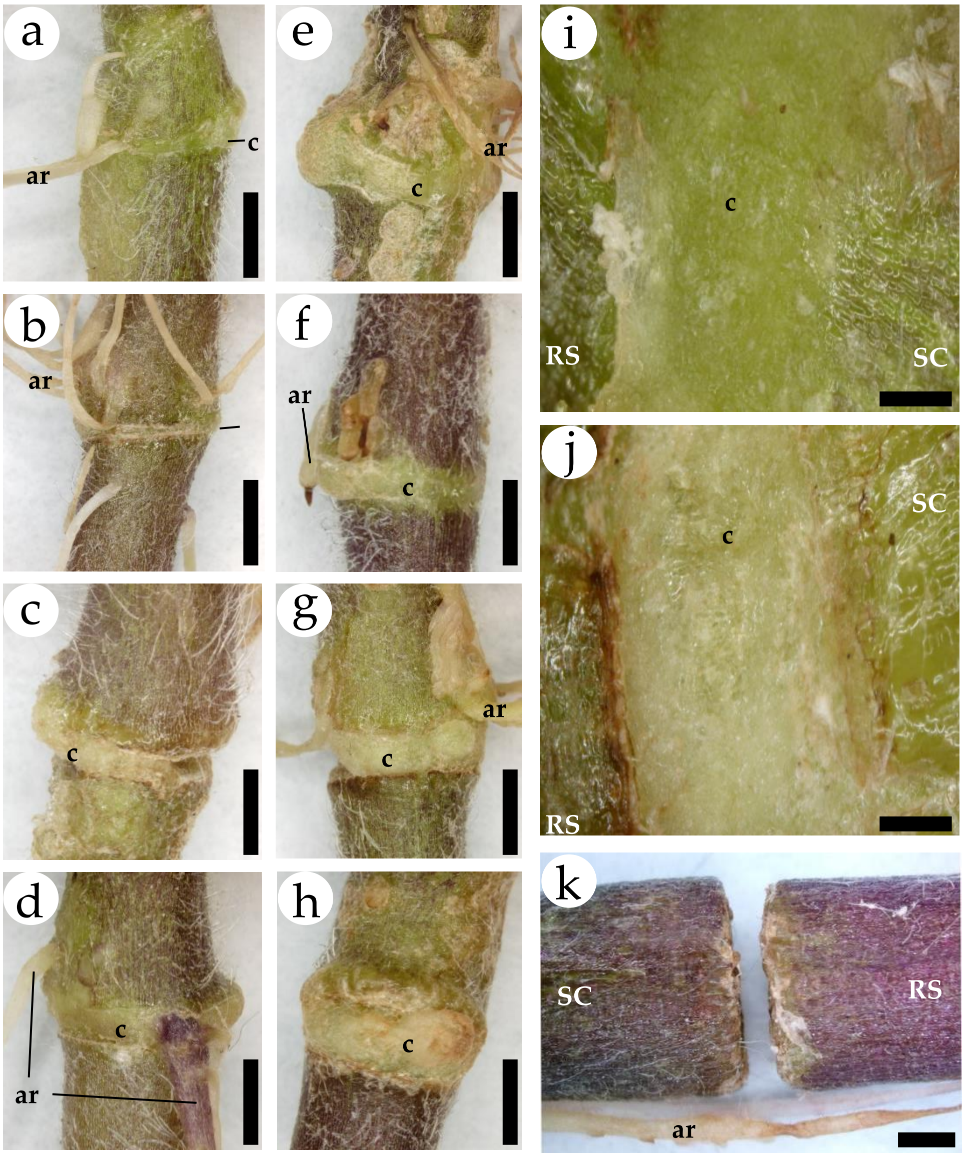

Figure S1. Graft junctions at (**a**–**d**) 10 and (**e**–**k**) 20 DAG. **a**, **b**, **e**, **f**, **i**, **k** Homograft; **c**, **g**, **j** Minibel (scion) – Marmande VR (rootstock) heterograft; **d**, **h** Marmande VR (scion)–Minibel (rootstock) heterograft. Scale bars: **a**–**h**, **k** = 1 mm; i, j = 0,5 mm. *ar* adventitious roots, *c* callus, *RS* rootstock, *SC* scion. In **a**–**h** the scion is the top part and rootstock the bottom part. Note the adventitious roots arising on the scion and the changes in the callus development. **i**, **j** Callus detail. **k** Unsuccessfully grafted plant, note the absence of scion – rootstock adhesion.

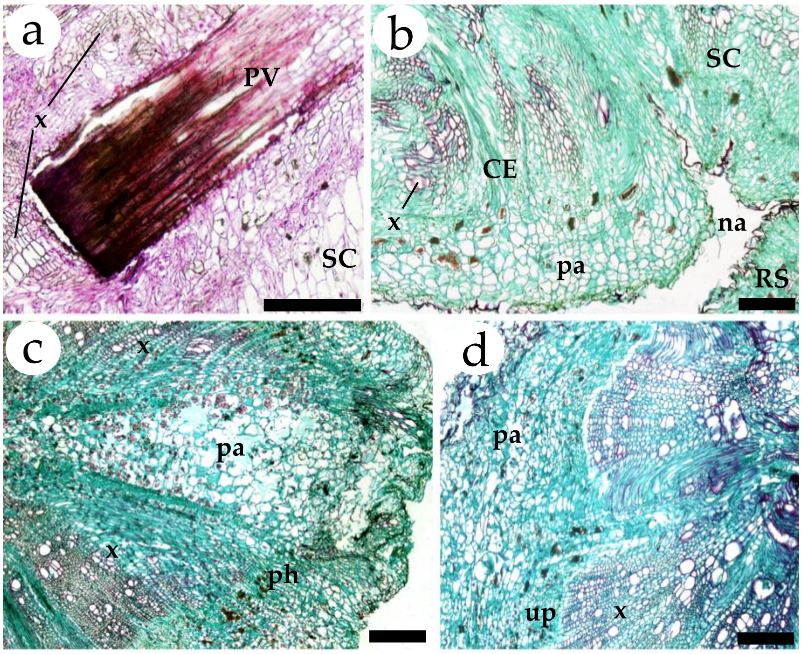

Figure S2. Details of graft histological development. **a** Pre-existing vascular tissue (PV) at 10 DAG; note how the most distal end is composed of dead cells only. **b** Longitudinal section of the expansion of the callus (CE) at 20 DAG; note the xylem (x) and the parenchyma (pa) towards the exterior. **c** and **d** Vestiges of the graft process at 210 DAG: **c** Transverse section of the graft junction area, showing the discontinuity of the vascular ring. **d** Transverse section of the graft junction area; note the anomalies of the vascular ring. a Haematoxylin-Eosin; **b**–**d** Safranin-fast green. **a**–**d** Bright field view. *CE*, callus expansion, *na* non-adhesion, *pa* parenchyma, *ph* phloem, *PV* pre-existing vascular tissue, *RS* rootstock, *SC* scion, *x* xylem. Scale bars: **a**, **d** = 100 μm; **b**, **c** = 200 μm.

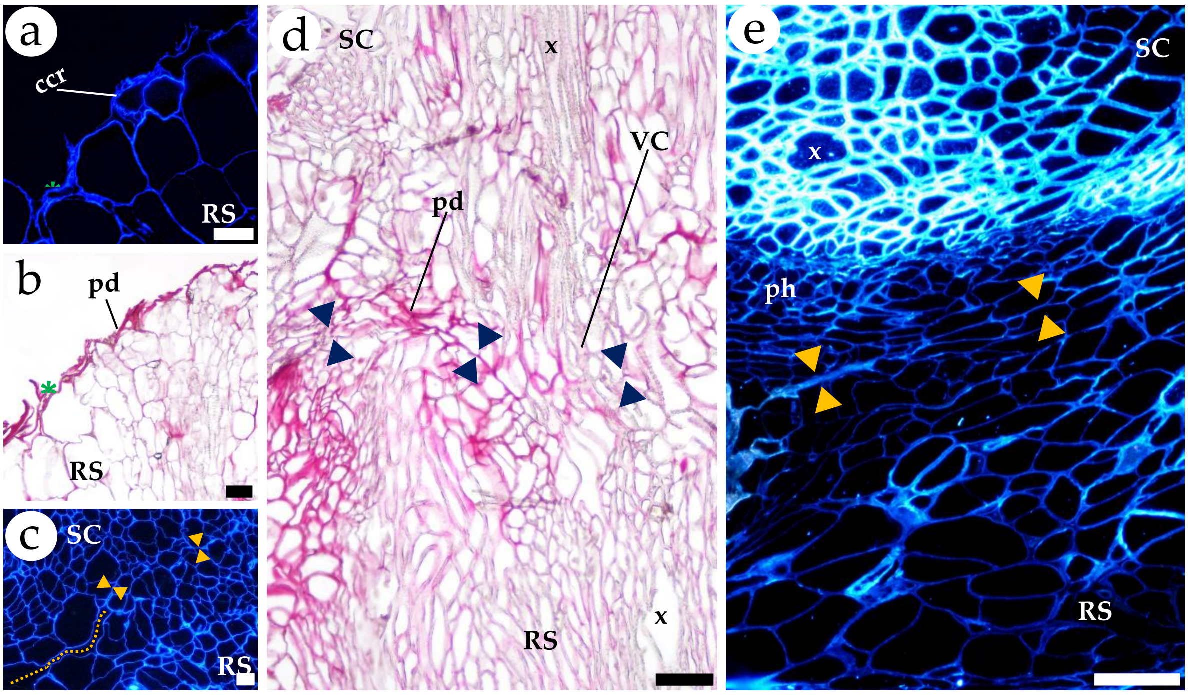

Figure S3. Longitudinal sections staining for polysaccharides (calcofluor White -cellulose- and ruthenium red -pectins-) from homografts. **a** Rootstock section at 2 DAG; note the cut edge, especially the crushed cells and remnants (ccr), as well as the intense fluorescence from cellulose of these cell walls. **b** Rootstock section at 4 DAG; note the deposition of high amount of pectins at the cut edge. **c** Early graft union section at 8 DAG; note the line of adhesion with cellulosic cell wall thickenings. **d** Graft union section at 20 DAG; note the permanence of the pectin depositions at the adhesion line as well as vascular (re)connections (*vc*). **e** Graft union section at 20 DAG; note the cellulosic cell walls at line of adhesion and transversal section of vasculature (xylem and phloem). **a**, **c**, **d** Calcofluor White; **b**, **e** Ruthenium red. **a**, **c**, **d** Epifluorescence microscopy. **b**, **e** Bright field view. *ccr* crushed cells and remains, *pd* pectin deposition, *ph* phloem, *RS* rootstock, *SC* scion, *VC* vascular connections, *x* xylem. Green asterisk indicates cut edges. Triangle arrows indicate adhesion line. Scale bars: **a**, **c** = 50 µm; **b**, **d**, **e** = 100 µm.
